# A dataset describing chip parameters for rock breaking by chisel pick under deep-sea hydrostatic pressure

**DOI:** 10.1038/s41597-024-03419-5

**Published:** 2024-06-04

**Authors:** Zenghui Liu, Rui Lv, Xinlei Chen, Kai Liu, Peng Wu, Changyun Wei

**Affiliations:** 1 Engineering Research Center of Dredging Technology, Hohai University, Changzhou, 213022 Jiangsu China; 2 College of Mechanical and Electrical Engineering, Hohai University, Changzhou, 213022 Jiangsu China; 3 Department of Engineering Science, University of Oxford, Parks Road, Oxford OX1 3PJ UK; 4 Changjiang Nanjing Waterway Engineering Bureau, Nanjing, China

**Keywords:** Civil engineering, Mechanical engineering

## Abstract

Chip is a visual representation of rock breaking by cutter, and their related parameters are crucial for revealing the rock breaking mechanism in deep-sea mining. Based on sieving and three-dimensional size measurement methods widely used in mining engineering, this paper reports a dataset of chip parameters for rock breaking by chisel pick under deep-sea hydrostatic pressure. Specifically, we first designed an experimental setup that can accurately simulate deep-sea hydrostatic pressure, conducted rock breaking experiments and carefully collected chips. Subsequently, those chips were sieved, high-resolution images were collected, and the coarseness index (*CI*), chip size uniformity (*n*), absolute chip size (*d*_e_), and fractal dimension (*D*) were measured. Finally, three-dimensional size (long, intermediate and short) was measured for 3064 chips with particle sizes greater than 4.75 mm. This dataset will be used by researchers to validate numerical simulations or optimize equipment structures related to deep-sea mining, including deep-sea rock mechanics, mining cutter and conveyor pipes.

## Background & Summary

The marine environment hosts a diverse range of mineral resources, encompassing not only traditional oil and natural gas, but also various underrecognized and underutilized minerals^1^. As the demand for mineral resources continues to grow while land-based resources become increasingly depleted, the exploration of seabed resources has emerged as a pivotal strategy to alleviate the global resource deficit. The deep-sea, typically situated 200 meters below sea level, represents one of the largest and most challenging-to-access biological habitats on Earth. Nonetheless, deep-sea mining has attracted global attention, prompting national mining companies and scientific research institutes to pursue reasoned approaches to deep-sea mining^2,3^. Currently, the predominant mining method for rock-based polymetallic sulfides (PMS) is the Pipe-Lift Mining System, which involves mining vehicle, pipe, and sea support vessel^4^. It is worth noting that research on rock fragmentation in the deep-sea environment plays a critical role in enhancing both mining efficiency and subsequent processes. Given the importance of preserving the delicate ecological balance during deep-sea mining operations^5^, the conventional blasting method is deemed unsuitable. On the contrary, mechanical mining is considered more feasible. During the process of rock breaking with mechanical cutting tools, a large amount of mineral chips is generated^6^. Nevertheless, the deep-sea hydrostatic pressure imposes constraints on large-scale rock extraction^7,8^.

Research on rock breaking under hydrostatic pressure is still in its primary stages. In order to comprehensively explore the mechanisms, accurate simulation of rock breaking under hydrostatic pressure is essential. However, the inherent complexity of deep-sea environment presents significant challenges in obtaining well-constrained field-scale rock breaking datasets. Consequently, meticulously controlled and reproducible laboratory experiments play a pivotal role in understanding and validating the process of rock breaking under hydrostatic pressure. Rock chip is an apparent phenomenon of rock breaking by cutter^9^. Parameters associated with rock chips, such as the coarseness index (*CI*), chip size uniformity (*n*), absolute chip size (*d*_e_), and fractal dimension (*D*), offer valuable insights into the mechanisms and efficiency of rock breaking^10–12^. These parameters are instrumental in validating the rationality of simulations^13,14^. Therefore, the study of chip parameters is well-established in land geotechnical engineering contexts, including mining^15^, tunneling^16^, and dredging^17^ processes. Furthermore, in the context of conveying broken minerals through pipelines using pneumatic or hydraulic methods, mineral size plays a critical role^18^. For instance, The Japan Oil, Gas and Metals National Corporation (JOGMEC) conducted successful tests for pipeline conveying of deep-sea broken minerals, focusing on conveying a maximum mineral size of 30 mm^19,20^. As such, the chip parameters resulting from rock breaking under hydrostatic pressure hold significance for the design and optimization of conveying pipelines. Unfortunately, detailed experimental data pertaining to rock breaking chip parameters under hydrostatic pressure are scarce due to the high costs associated with deep-sea hydrostatic pressure simulation experimental platforms. Therefore, conducting rock breaking experiments under hydrostatic pressure to obtain meaningful data on chip parameters in laboratory settings is paramount. This is particularly rare in the field of deep-sea mining, especially with experiments conducted under authentic hydrostatic pressure. As a result, an experimental dataset describing chip parameters for rock breaking holds substantial value.

Based on this background, the Engineering Research Center of Dredging Technology of Hohai University has designed a deep-sea hydrostatic pressure rock cutting experimental platform. This innovative platform can precisely replicate rock breaking at various cutting depths under hydrostatic pressure. As a result of this technological advancement, the rock breaking chip parameters have been meticulously compiled into a dataset, encompassing essential metrics such as *CI*, *n*, *d*_e_, *D* and three-dimensional size (long, intermediate and short). This dataset will offer invaluable experimental data for understanding the variation of rock chip parameters under hydrostatic pressure. Furthermore, it can be utilized to validate rock breaking simulations, enhance deep-sea rock mechanics theories, and optimize pipeline lifting systems for the advancement of deep-sea mining equipment.

## Methods

### Rock sample and chisel pick

**Fig. 1:**
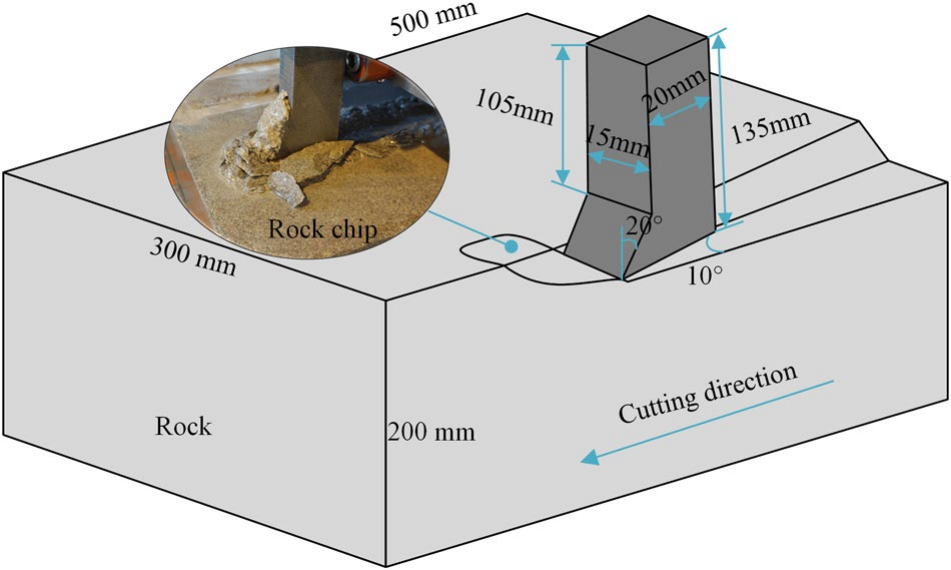
Geometry of the sample and chisel pick.

The sandstones utilized in the experiments, as depicted in Fig. 1, were sourced from the same mining area and uniformly cut to dimensions of 500 mm × 300 mm × 200 mm. Following measurement, it was determined that the rock surfaces exhibited favorable flatness and isotropy, ensuring consistent cutting depth during the rock cutting process with the chisel pick. The chisel pick, a commonly employed rock-breaking tool, was geometrically crafted via wire cutting to meet specified parameter sizes. Due to its exceptional rock-breaking capabilities, the chisel pick is widely utilized in mineral resource mining, prompting numerous researchers to investigate its rock-breaking characteristics and mechanisms^21–24^. The performance of both the rock and the chisel pick underwent testing, and the findings are summarized in Table 1. The rock mechanics tests were performed in accordance with the available ASTM standards^25^. The material chosen for the chisel pick is high-speed steel, the mechanical properties of which are based on standardized test methods for common metallic materials^26,27^.

**Table 1:** Summary of the samples and cutter properties.

Rock sample		Cutter	
Type	Sandstone	Type	Chisel pick
Compression strength (MPa)	24.6	Category	HSS (W6Mo5Cr4V2)
Tension strength (MPa)	2.2	Hardness HRC	65
Elastic modulus (Gpa)	2.57	Bending strength (GPa)	3.9
Density (g/mm^3^)	2.22	Impact Toughness (MJ/m²)	0.7
Poisson ratio	0.32	—	—

### Experimental set-up

**Fig. 2:**
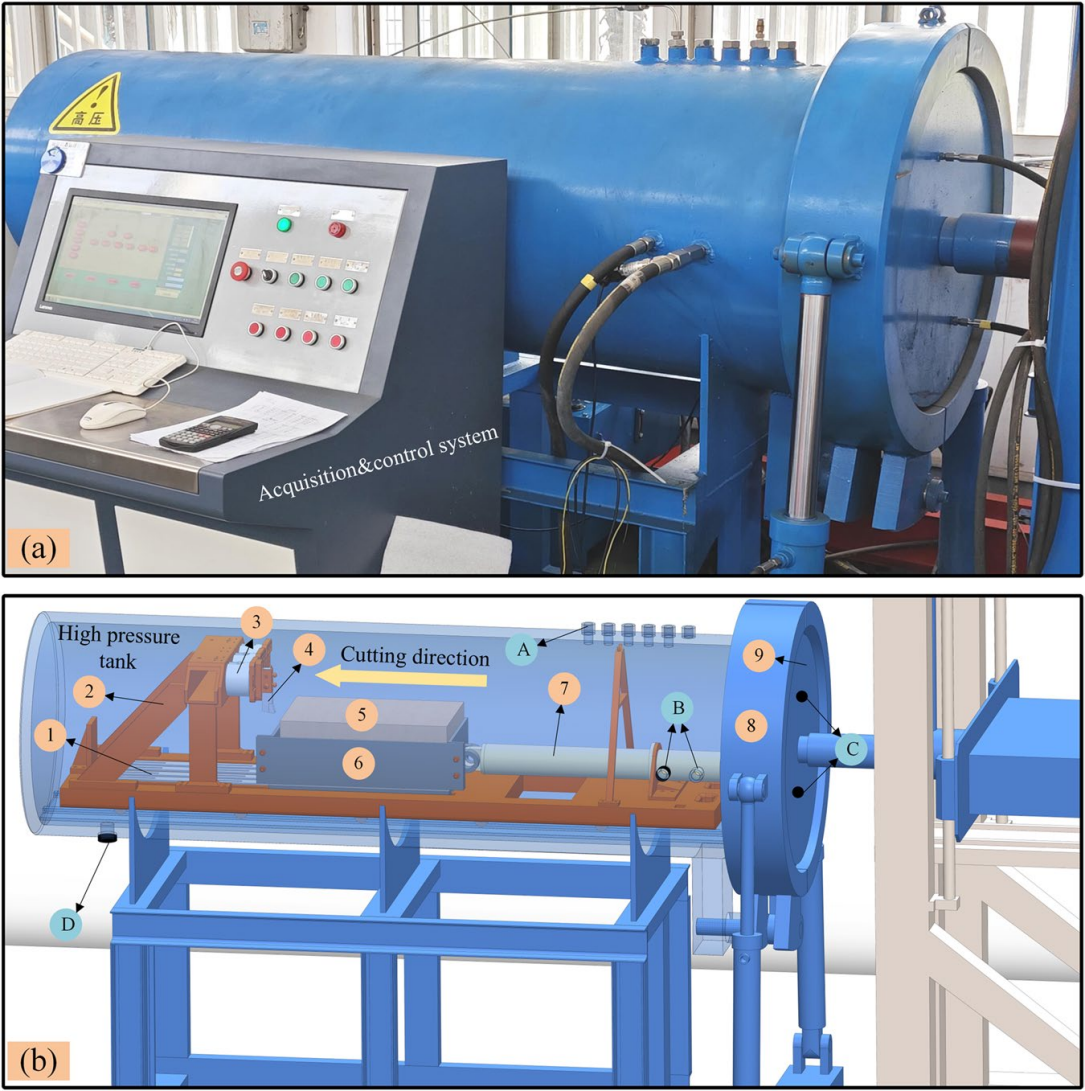
Hydrostatic pressure rock breaking experimental platform and physical model. Numbers 1 to 9 and letters A to D denote rock cutting device structure, sealing devices and interface respectively. Nylon strips 1; Cutter holder 2; Cutter base 3; Chisel pick 4; Rock 5; Rock box 6; Propulsion cylinder 7; Arm 8; Hatch 9. Adding water inlet (A); Propulsion cylinder inlet and outlet (B); Hydrostatic pressure increase and decrease (C); Drainage outlet (D).

The main components of the deep-sea hydrostatic pressure rock cutting experimental platform are rock cutting system, acquisition&control system and hydrostatic pressure loading system. Its main structure is shown in Fig. 2.

The rock cutting device is composed of several key elements, including the chisel pick, cutter base, cutter holder, rock, rock box, nylon strips, and propulsion cylinder. The chisel pick is affixed to the cutter base and can be readily adjusted to the desired cutting position. The rock is securely positioned within the rock box, with the cutting depth adjustable by adding varying thicknesses of steel plates to the rock box. The cutting motion is initiated by the propulsion cylinder, which is powered by the hydraulic pump station. Nylon strips are placed beneath the rock box to diminish friction resistance during linear movement. The rock cutting equipment is designed with pulleys at the base, enabling rapid entry and exit through the guide rails inside and outside the high-pressure tank.

Effective sealing of the high-pressure tank is achieved through the collaboration of the hatch and arms. When the rock cutting device is pushed into the high-pressure tank, the control system orchestrates the hatch to connect with the high-pressure tank, after which the arms close. Throughout the rock cutting process, the data acquisition and control system gathers cutting speed and hydrostatic pressure parameters, ensuring the stable operation of the cutting process.

**Fig. 3:**
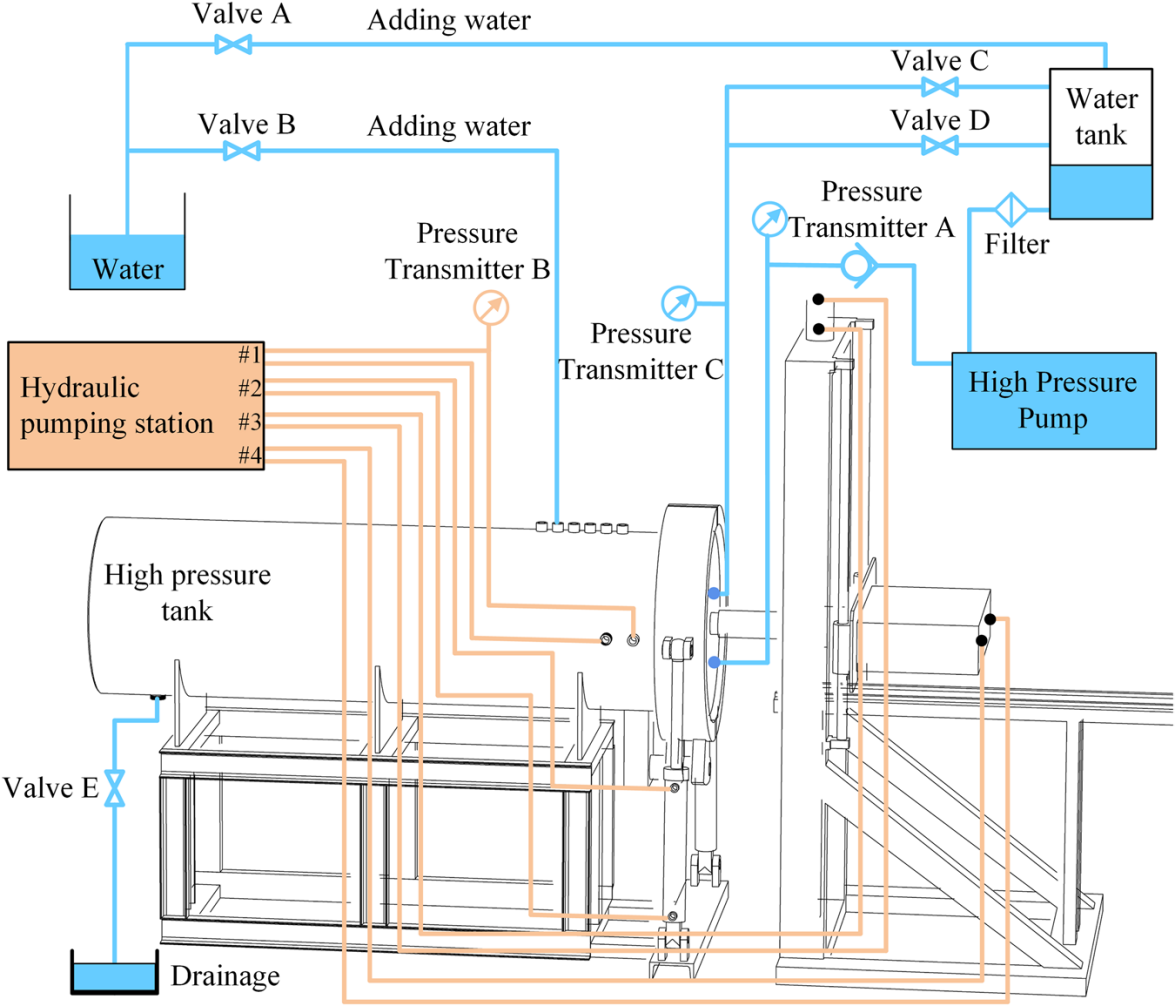
Schematic figure of the hydrostatic pressure loading system: details of valves, sensors and pump position.

The operational principles underlying both the hydrostatic pressure loading and rock cutting motion are visually depicted in Fig. 3. The hydraulic pump station propels the pertinent structures through components #2, #3, and #4 to achieve high-pressure tank sealing. The hatch incorporates multiple layers of seals, permitting hydrostatic pressures of up to 20 MPa within the high-pressure tank. Hydrostatic pressure is generated using a high-pressure pump. During the hydrostatic pressure loading process, valves C and D are closed, and valve B is opened to fill the tank with water before being closed, Valve E is the drain port and is also closed during the experiment. Subsequently, the high-pressure pump injects water into the high-pressure tank, causing an increase in pressure. If the water level in the tank is low at this time, valve A is opened to ensure timely replenishment to maintain a high water level.

As the high-pressure tank’s internal hydrostatic pressure reaches the predetermined value, the system enters the pressure stabilization stage. During this phase, the control system dynamically adjusts the pressure relief valve C based on real-time pressure readings from sensors A and C, allowing for the discharge of water from the high-pressure tank to the water tank, thus achieving dynamic stabilization of hydrostatic pressure. Once hydrostatic pressure is stabilized, hydraulic pump station #1 connects to the rock cutting device inside the tank via the external interface of the high-pressure tank, thereby initiating the rock cutting process. It is important to note that the extension of the hydraulic actuator occupies space, potentially causing a surge in hydrostatic pressure within the high-pressure tank. Consequently, during the rock cutting phase, relief valve D is opened to balance the surge in hydrostatic pressure.

Upon completion of the experiment, the control system fully opens pressure relief valve C to reduce the hydrostatic pressure to a safe level. Following this, valve E is opened to drain the water from the high-pressure tank. To ensure the stable operation of the hydrostatic pressure loading system, Table 2 provides a comprehensive summary of the technical parameters of the main components within the hydrostatic pressure loading system.

**Table 2:** Specifics of hydrostatic pressure loading system.

Components	Specifics
High pressure pump	Type: Italy AR
High pressure tank	Outer diameter: 1400 mm; Inner diameter: 1000 mm; Length: 3000 mm
Valve A, B and E	Type: High Pressure Needle Valve; Maximum Pressure: 42 MPa
Valve C	Type: Pressure Relief Valve; Maximum Pressure: 50 MPa
Valve D	Type: Relief Valve; Regulating Range: 0~35 MPa; Flow Rate: 40 L/min
Pressure transmitters	Range: 0~30 MPa; Output signal: 4~20 mA; Accuracy level: 0.1% FS; Power supply: 24 VDC
Water tank	Volume: 100 L
Pipeline	Inner diameter: 19 mm; Maximum pressure resistance: 70 MPa

### Experimental protocol

The experiments involved varying cutting depths (3 mm, 6 mm, 9 mm, 12 mm, and 15 mm) and hydrostatic pressures (3 MPa, 6 MPa, 9 MPa, and 12 MPa). To comprehensively characterize the rock-breaking chip parameters under hydrostatic pressure, corresponding cutting depth experiments under atmospheric pressure were also conducted. These specific experimental parameters effectively capture the variation of rock-breaking chip parameters under hydrostatic pressure in deep-sea conditions. The complete experimental process encompasses rock breaking, chip collection, sieving, and measurement.

#### Rock breaking under hydrostatic and atmospheric pressure

**Fig. 4:**
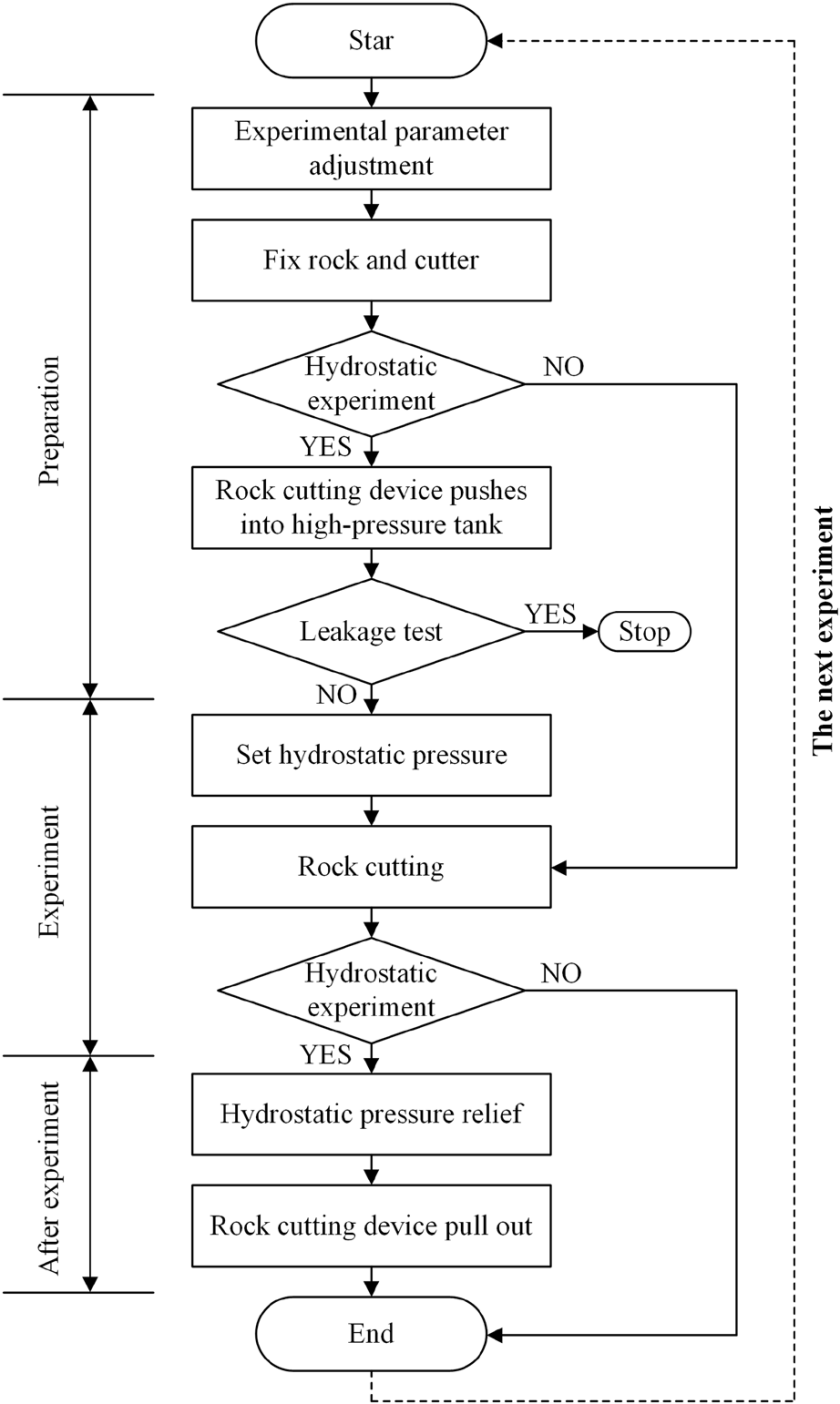
Rock breaking experimental procedure. (**a**) Adjust the experimental parameters required in this experiment, including cutting speed and cutting depth. The cutting speed is set using the electromagnetic speed control valve, which ensured that the cutting speed remained stable during the experiment, and all experimental data in this dataset are conducted at this cutting speed. The cutting depth can be adjusted by using different thicknesses of steel plates in the rock box. (**b**) Fixing the rock and chisel pick. A sample of processed rock is selected and placed in the rock box of the rock cutting device, and the rock is secured using side plates to prevent movement of the rock during the cutting process. Similarly, the chisel pick is fastened to its holder and ensures that there is no lateral displacement. Due to the presence of the steel plate at the bottom of the rock, the distance between the fastened chisel pick and the rock surface is exactly the required cutting depth for this experiment. (**c**) The experiment consisted of rock cutting under both hydrostatic pressure and atmospheric pressure. For rock cutting under hydrostatic pressure, after completing steps a and b, it is necessary to push the rock cutting device into the high pressure tank, and then close the hatch and arm to complete the sealing of the end of the high pressure tank. For rock cutting under atmospheric pressure, the rock cutting can be done directly in step f because there is no need to set the hydrostatic pressure or other operations. (**d**) Pressure relief test. Close the drain valve, open the water inlet, and fill the high pressure tank with water. Since the high pressure experiment requires a high degree of sealing, it is also necessary to pressure test the high pressure tank before conducting the hydrostatic pressure rock cutting experiment. During the test, once there is a leakage of water to stop the experiment, carefully check the place of pressure relief, and ensure that the tank leakage does not occur again. (**e**) Setting the experimental hydrostatic pressure. Use the control system to set the required hydrostatic pressure for the experiment. After the hydrostatic pressure is set, click “Run” and the control system will continuously increase the high pressure tank hydrostatic pressure. After reaching the required hydrostatic pressure, the pressurization and depressurization reach a dynamic balance, and the hydrostatic pressure stabilizes within the set value. (**f**) Rock cutting. In the control system, turn on the hydraulic pump and control the hydraulic push rod to advance and complete the rock cutting. After the rock is broken, operate the hydraulic actuator backward to complete the retracting action. If the experiment is under hydrostatic pressure, steps g and f are also required. If the experiment is under atmospheric pressure, this experiment is finished. (**g**) Pressure relief of the high pressure tank. Although the rock cutting experiment under hydrostatic pressure is completed, but at this time there is still high pressure water inside the tank. Using the control system, click on the “pressure relief”, the system will be gradually stepped pressure relief, when the tank internal pressure is low, open the drain valve, drain the tank of water. (**h**) Rock cutting device push out. The rock cutting device is pushed out and cleaned of residual water by opening arms and hatch. The rock cutting device is then adjusted in preparation for the next experiment.

The detailed steps of rock breaking under different pressures are summarized as Fig. 4.

#### Rock breaking chip collection, sieving and measurement

**Fig. 5:**
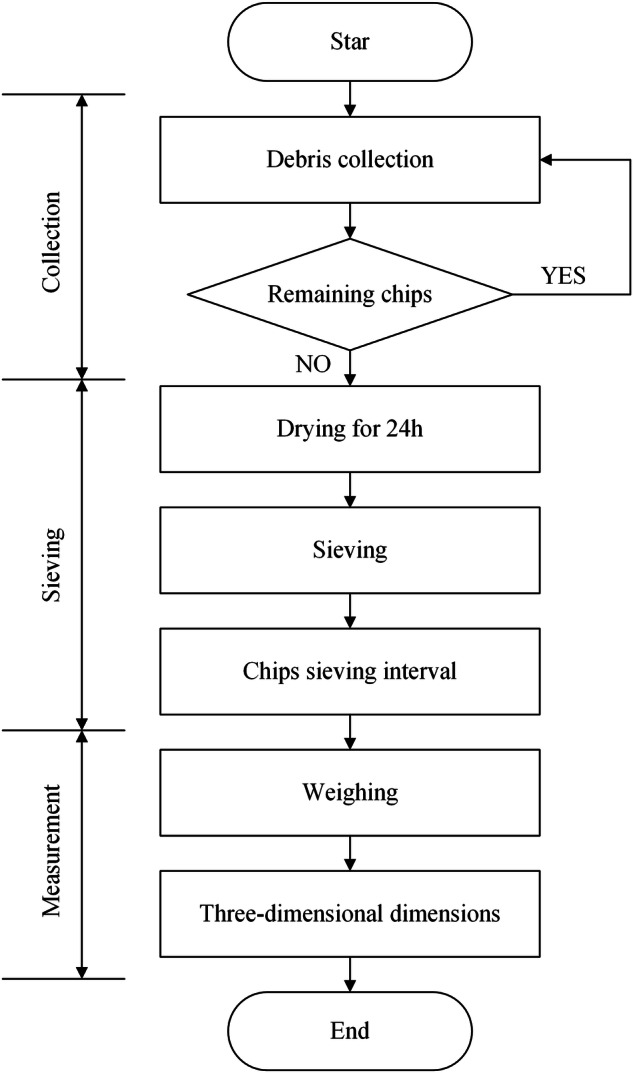
Chip handling process.

To ensure the accuracy of the chip analysis, we divide the chip handling process into three steps, including collection, sieving and measurement, as shown in Fig. 5. The chips from each rock breaking experiment need to be carefully collected and placed in clear bags labeled with serial numbers. In a hydrostatic pressure environment, all the rock chips will collect in the cutting path due to the resistance limitations of the water surrounding the rock. While under atmospheric pressure, some of the chips will be ejected. In order to collect the chip better, we added a transparent shield around the equipment. Experiments show that the shield can confine all the falling chips to a smaller area and improve the collection efficiency.

**Fig. 6:**
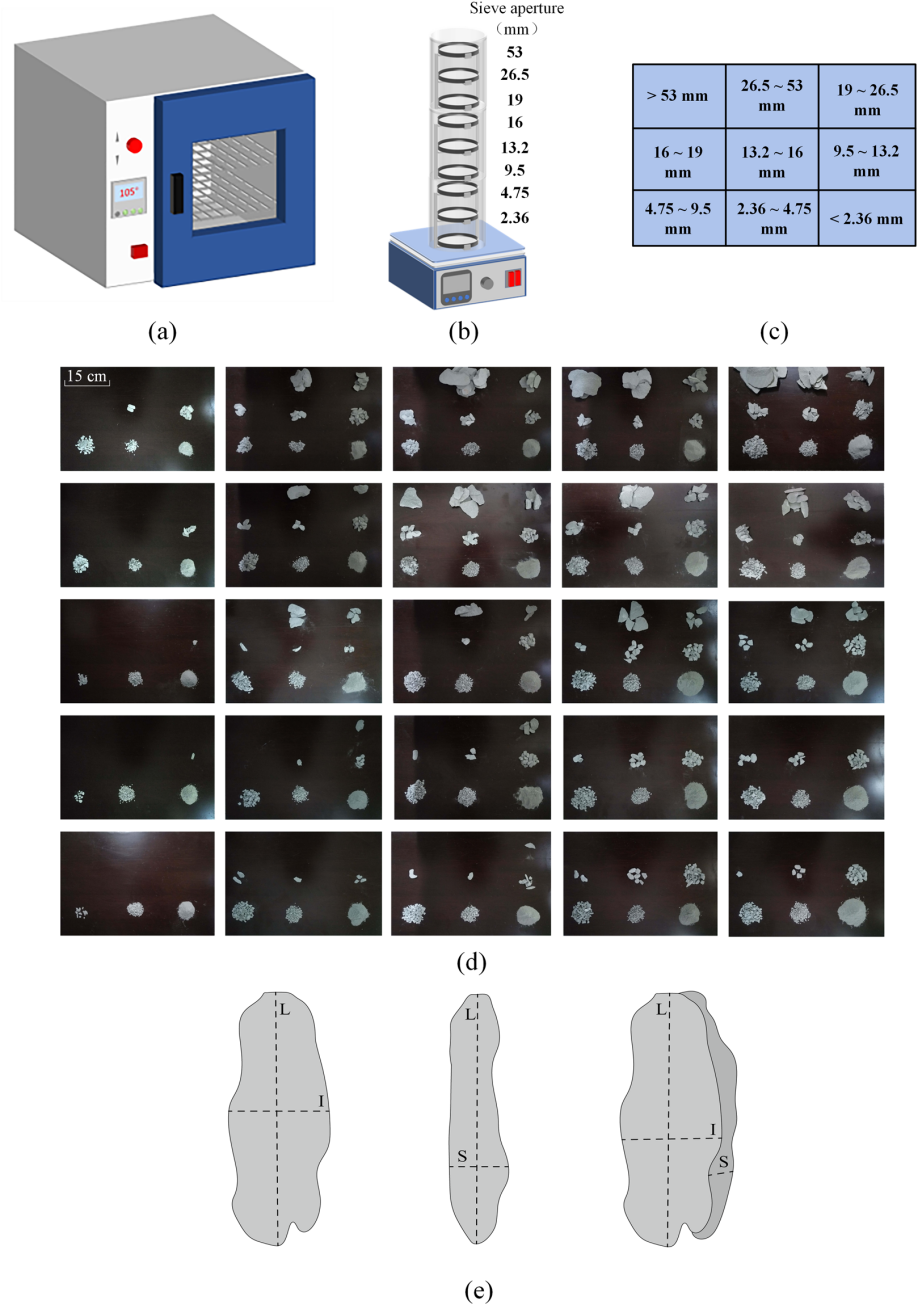
Chip sieving and measurement.

Upon completion of all experiments, the collected chips undergo a sieving process illustrated in Fig. 6. Given the high water content of the collected rock chips, they require drying to facilitate effective sieving. When the drying temperature is too low, the chips cannot dry effectively, increasing the drying cycle. When the temperature is too high, the high temperature will cause damage to the chips due to rapid drying, which is not conducive to the measurement of the chips. To prevent secondary damage to the chips from high temperatures, the oven temperature is maintained at 105 °C during a 24-hour drying period, as depicted in Fig. 6(a). Subsequently, nine sieve apertures are selected, and the rock chips are sieved for five minutes utilizing a sieving machine with a vibration frequency of 120 Hz, as shown in Fig. 6(b). The sieved chips are then categorized into nine intervals based on the aperture of the screen mesh, as evidenced in Fig. 6(c). Figure 6(d) provides insight into the distribution of chips within the nine intervals under each experimental scenario post-sieving, effectively reflecting chip variations under different hydrostatic pressures.

For the final chip measurement, an electronic scale is employed to weigh and record chips in each interval. Following this, the three-dimensional size (long, intermediate, short) of the chips are measured using digital calipers, as indicated in Fig. 6(e). Weighing of chips is conducted for all chip size intervals, while the measurement of the three-dimensional size is specifically carried out for 3064 pieces of chip with particle sizes exceeding 4.75 mm. This selection criterion is attributed to the fact that excessively small chips cannot be accurately measured and do not sufficiently represent the morphological changes of rock chips under hydrostatic pressure.

### Chip analysis

Analysis of chip size and shape has been widely used in many scientific and engineering studies to determine the fundamental properties of chip and their effects on specific processes. Common methods for analyzing chip parameters are described below.

#### Coarseness index

The coarseness index (*CI*) is a convenient test method for analyzing the distribution of chips and their size. *CI* was utilized by Barker^28^ in engineering techniques. In his earlier comparative study, he found that a higher *CI* value indicates a larger block of chip. It is calculated by summing the cumulative weight percentage of rock chip retained on each sieve through the sieve mesh. The *CI* value is calculated by Eq. (1) and is a dimensionless parameter.

1 $$CI=100\mathop{\sum }\limits_{{\rm{i}}=1}^{{N}_{c}}\frac{{M}_{i}}{{M}_{T}}$$

where *M*_*i*_ is the weight of rock chip whose size is larger than sieve aperture *r*_*i*_, *M*_*T*_ is the total weight of chip for this sieving, *N*_*c*_ is the number of sieve type used.

#### Rosin-rammler function

The Rosin-Rammler method is often used to analyze the generation of rock breaking & cutting in the tunneling and mineral processing industries^29^. The distribution of rock chips can also be examined by using the function.The Rosin-Rammler function describes the mass distribution function as an equation in exponential form. The Rosin-Rammler equation is stated as Eq. (2).

2 $$\log \left[ln\left(\frac{100}{R}\right)\right]=n\,\log \,r-n\,\log \,{d}_{e}$$

Where, *R* (%) is the cumulative mass retained on the sieve aperture *r*_*i*_, *d*_e_ (mm) is the chip size parameter defined as the chip size when *R* = 36.79% (by weight). *n* is the chip size distribution parameter defined as rock chip size uniformity. Therefore *n* represents the degree of size difference between chips, the larger *n*, the more uniform size between the debris. The calculation of *n* and *d*_e_ can be plotted in the form of a linear function of Eq. 2, the slope is *n* and the intercept are -*n*log*d*_e_.

#### Fractal function

Fractal geometry was originally used to describe highly irregular and self-similar objects^30^. The macroscopic fragmentation of rock materials under loading is characterized by a small group of chips, while smaller fractures consist of smaller cracks evolving and aggregating, and this similar behavior leads to self-similarity of chip. Therefore, fractal theory have been applied to the field of rock fragmentation and a lot of researches have been carried out. The fractal theory is utilized to calculate the fractal dimension (*D*) of the fragments demonstrated as shown in Eq. (3).

3 $$ln\left(\frac{{M}_{{\rm{r}}}}{{M}_{{\rm{T}}}}\right)=(3-D)\,ln\left(\frac{r}{{r}_{\max }}\right)$$

Where, *M*_*r*_ is the cumulative mass of rock chip passing through a given sive aperture *r*, *M*_T_ is the total mass of chips, and *r*_max_ is the maximum sive aperture. It can be seen that Eq. (3) is a linear function with a slope factor of 3-*D*. Therefore, based on the sieving results of the rock chip, the fractal dimension *D* can be obtained. The fractal dimension reflects the degree of rock breaking, the larger *D*, the higher proportion of small chip sizes.

**Fig. 7:**
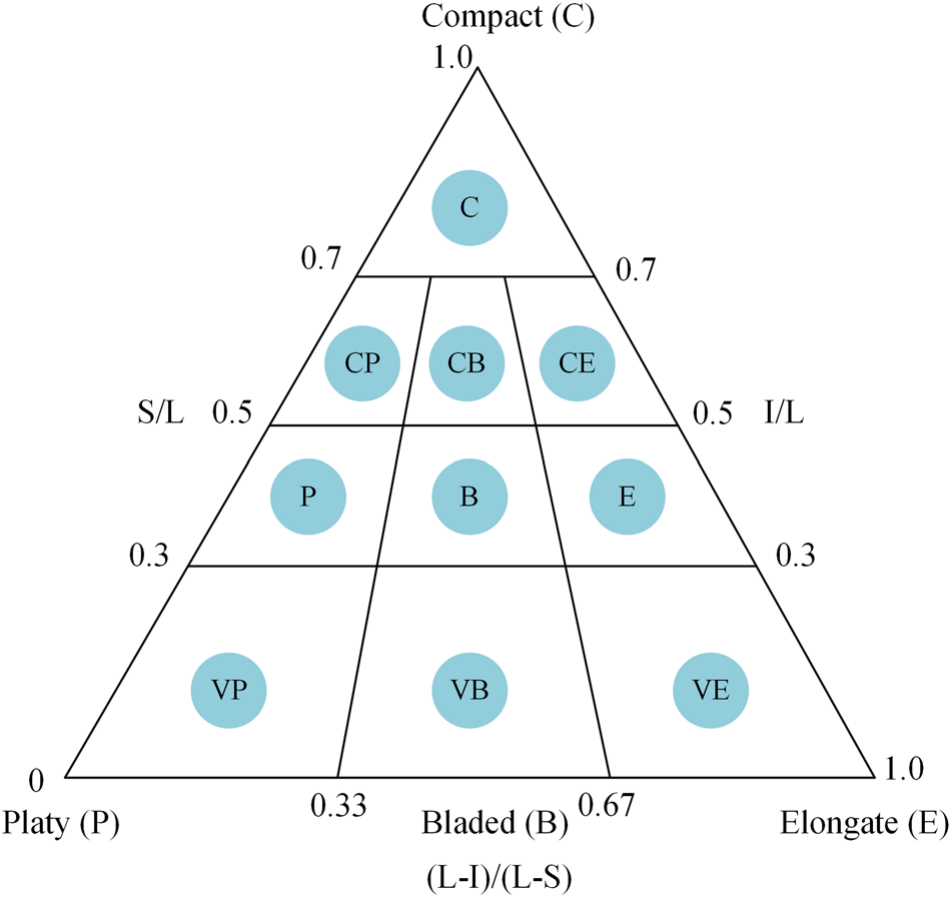
10 classes in triangle diagram: C, Compact; CP, Compact-Platy; CB, Compact-Bladed; CE, Compact-Elongate; P, Platy; B, Bladed; E, Elongate; VP, Very Platy; VB, Very Bladed; VE, Very Elongate. (after zingg^31^, Sneed^32^ and Mohammadi^29^).

*Chip shape* Particle shape is another fundamental property that can provide important information about rock chip. As with particle size, the shape of minerals and rock chip may be determined by a variety of factors, such as an expression of the overall appearance of the particle, the aspect ratio of the particle; roundness, the degree of roundness or angularity of the edges of the particle; and sphericity, a measure of how closely the shape of the particle approximates that of a true sphere. However, the shape of the chip cannot be measured visually, and several formulas are commonly used to determine the shape of the particles. In the dataset of this paper, Based on the three-dimensional dimensions (L, I and S) of the debris measured by the digital vernier calipers in Fig. 6(e), the ratios S/L, (L-I)/(L-S) and I/L were calculated and plotted in a ternary diagram. Finally, we categorized the shape of chip into 10 categories based on Zingg^31^, Sneed^32^ and Mohammadi^29^, as shown in Fig. 7.

## Data Records

The dataset is obtained from 10.6084/m9.figshare.25102847^33^. To facilitate quick access, the data storage structure is designed as shown in Table 3. The “README” folder that describe the structure, the information within each folder, and give information about the symbols and abbreviations used in the data tables. The “Sieving and Measuring” folder organizes the raw rock chip data of different experimental groups, including the weight of chip retained by different sive aperture and the three-dimensional size of chip with a sive aperture larger than 4.75 mm. The “Hydrostatic pressure” folder records the rock chips displacement and hydrostatic pressure changes under different hydrostatic pressure loading environments, which will facilitate the data users to inquire about the environmental factors of chip production. The “Chip shape” folder organizes the shape parameters of 3064 chips according to hydrostatic pressure. The “Photo” folder contains images of rock chip from each experimental group. The picture in the folder are named “P + H”, which represent the hydrostatic pressure (MPa) and cutting depth (mm), respectively. *CI*, *D*, *n*, and *d*_e_ describing the parameters of the chip were stored in .xlsx file format, respectively.

**Table 3:** Data Repository structure.

Data Repository structure	Format	Detail
README	Folder	Structure and information on each folder; Abbreviations used in dataset
Sieving and Measuring	Folder	Raw data on chip parameters
Hydrostatic pressure	Folder	Cutting displacement and hydrostatic pressure
Chip shape	Folder	Chip shape parameters
Photo	Folder	Pictures of chip sieving interval and shape
Coarseness index	.xlsx	Experimental data from *CI*
Fractal function	.xlsx	Experimental data from *D*
Rosin-Rammler function	.xlsx	Experimental data from *n* and *d*_e_

## Technical Validation

**Fig. 8:**
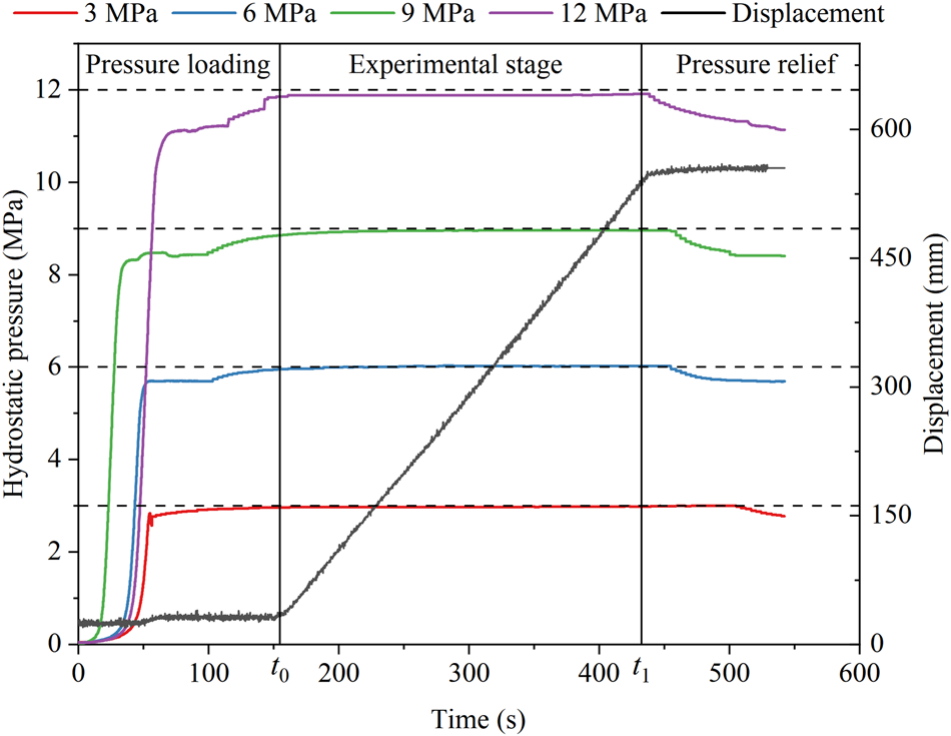
Hydrostatic pressure control during experiments.

The precise control of hydrostatic pressure plays a crucial role in ensuring the validity of the experimental data. Despite stable control of the hydrostatic pressure at the designated set value (*t*_0_ ~ *t*_1_) throughout all experiments, a minor fluctuation range was observed during the cutting process, as visually depicted in Fig. 8. Analysis of the measured hydrostatic pressure detection curves confirms that the hydrostatic pressure loading system adeptly manages the fluctuation range of the hydrostatic pressure, thereby meeting the essential experimental criteria.

**Fig. 9:**
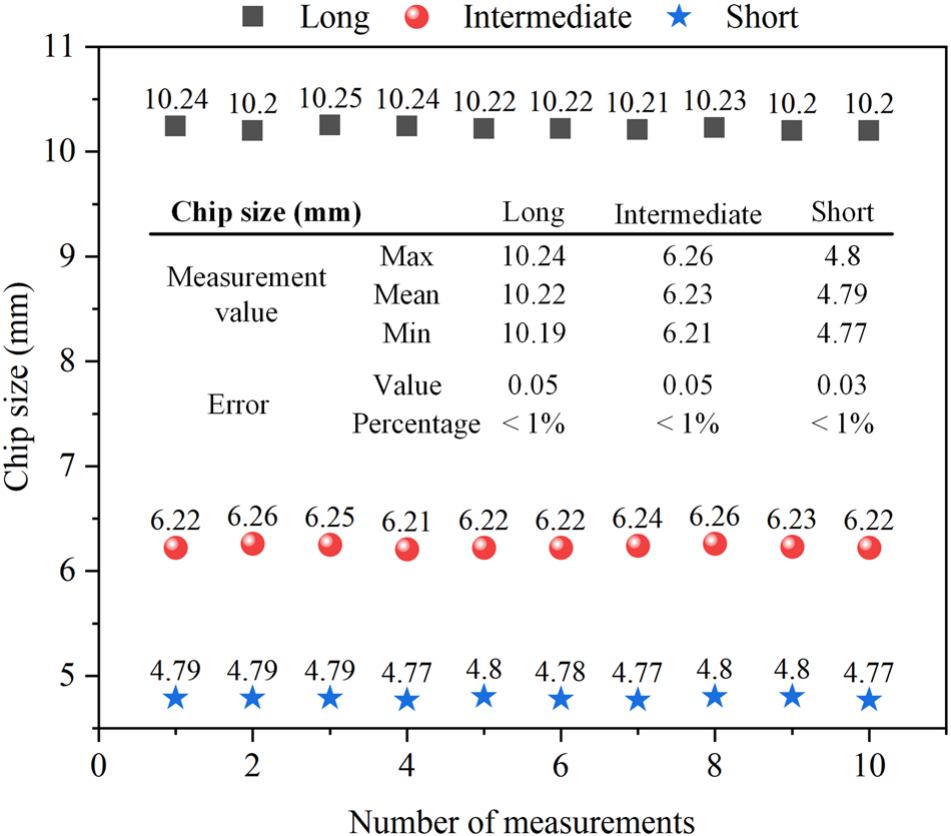
Accuracy of three-dimensional size measurement of the example chip.

A potential source of error in the experiment could have stemmed from the collection and measurement of rock chips. The process of cutting rock at atmospheric pressure is often accompanied by acoustic noise and the rapid ejection of large rock fragments. To mitigate this, a transparent shield was installed on the rock cutting device for containment of the generated rock chips. This method effectively controlled the collection of rock chips at atmospheric pressure, minimizing experimental errors. However, when the rock cutting device was introduced into the high-pressure tank for hydrostatic pressure loading, the rock chips produced were obstructed by the surrounding water and remained affixed to the rock surface. Following the conclusion of the experiment, no chips were found inside the autoclave across all experimental groups. Furthermore, the fine powder resulting from the cutting process adhered to the rock surface and necessitated drying before chip collection. It is plausible that a marginal amount of fine powder may have been lost during the collection process, although this was considered insignificant in comparison to the overall chip sample. The three-dimensional size of the chips was measured using digital vernier calipers with a precision of 0.01 mm. To evaluate test reproducibility, repeated measurements were performed, yielding differences of no more than 0.05 mm, which was deemed sufficiently accurate for three-dimensional chip size measurement. In this paper, the measurement of the three-dimensional size of the chips (long, intermediate and short) was standardized, so the measurement error is largely due to the measurement tool. Any piece of chip collected in the experiment was taken for 10 measurements and its three-dimensional size are shown in Fig. 9. The measuring tool used in the experiment is a digital vernier caliper with an accuracy of 0.01 mm, and the repeated measurements of the three-dimensional size of the chips using this tool show that the measurement errors of the three-dimensional size of chips are within 0.05 mm. Considering that the smallest chip size is 4.75 mm, this equates to an error of less than 1%.

**Fig. 10:**
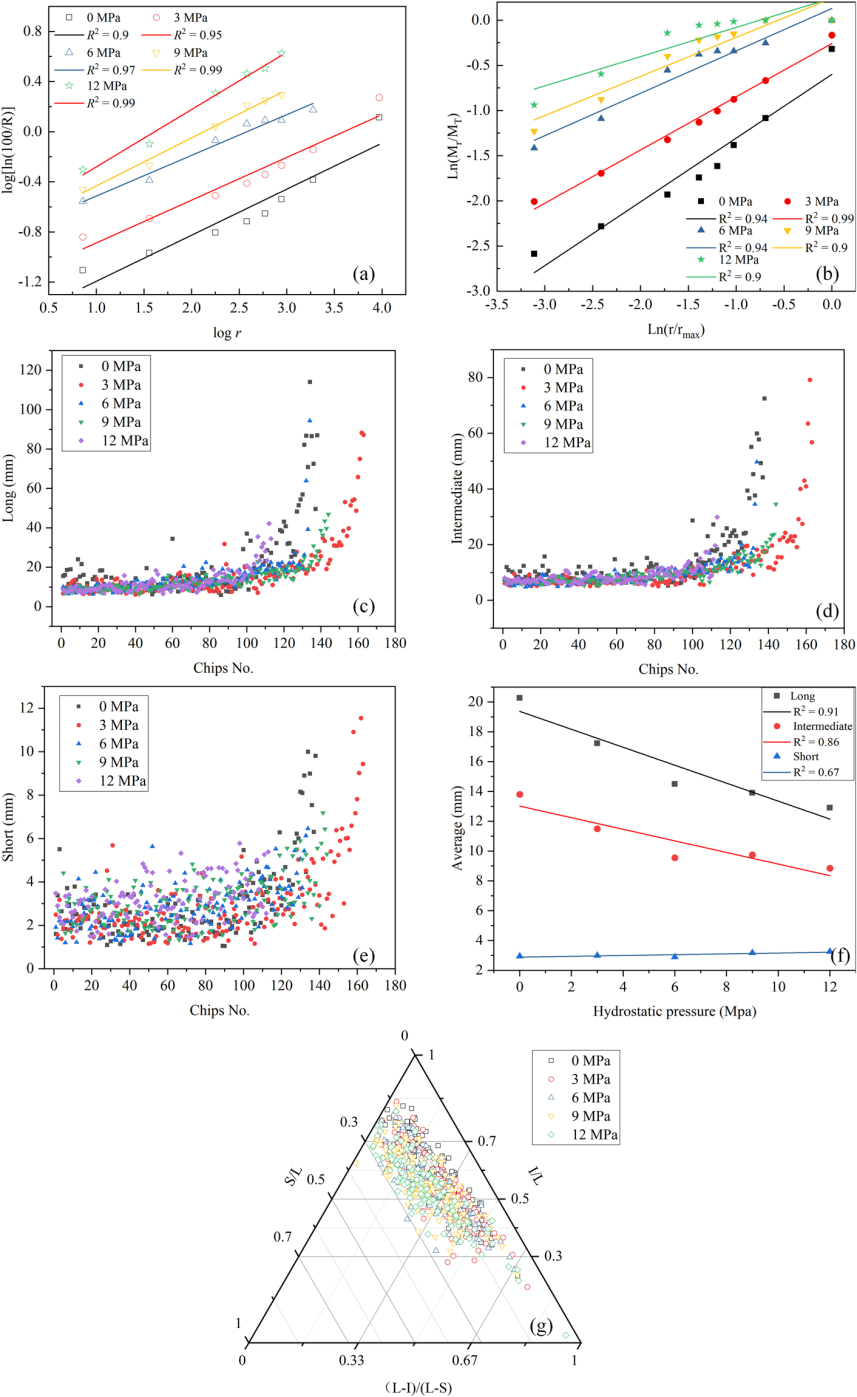
Example of data processing results of some chip parameters with different hydrostatic pressures at 9 mm cutting depth: (**a**) Rosin-Rammler function. (**b**) Fractal function. (**c**–**f**) Three-dimensional size and its average value. (**g**) Shape distribution.

Additionally, some of the chip parameter analyses were discussed in^7^ based on the dataset. Examples of chip parameters are shown in Fig. 10, providing meaningful insights into rock breaking chip parameters under hydrostatic pressure. Figure 10(a,b) represents the Rosin-Rammler function and Fractal function fitting results, respectively, which demonstrate a good linear relationship (R^2^ > 0.9) and can be well used for calculating chip size uniformity, absolute chip size, and fractal dimension. Figure 10(c–e) show the chip three-dimensional size for the raw data of the chip size and shape analysis. Figure 10(f) shows the three-dimensional average size under the influence of hydrostatic pressure, which exhibits a good relationship and further validates the data. Figure 10(g) shows the ternary plot of the chip shapes, which can well analyze the change and distribution of the chip shape under different hydrostatic pressures based on the criterion of chip shape delineation. Consequently, the conclusions drawn from the analyses affirmed the validity and reproducibility of the measurements.

## Usage Notes

The dataset can be utilized to calculate and analyze rock chip parameters, offering valuable insights for the design of deep-sea mining equipment such as pipes and cutterheads. Furthermore, it can contribute to a better understanding of deep-sea rock mechanics. The data can be analyzed in diverse ways to facilitate its use and further development. While this paper presents some common methods of analyzing the data, it can also be subject to statistical analysis for comprehensive exploration.

## Data Availability

The data in this paper was organized based on Microsoft Excel 2016 and the original data record format (.xlsx) was provided, which will make it easy for the user to import into any of the data analysis software such as Matlab, Python, and SPSS. No custom code was used in this study to organize or validate the dataset.
